# Supplementary figures and images for: Vitamin D receptor-deficient keratinocytes-derived exosomal miR-4505 promotes the macrophage polarization towards the M1 phenotype
Source: PeerJ. 2023 Aug 4;11:e15798. doi: 10.7717/peerj.15798 (PMC10405794; doi:10.7717/peerj.15798)

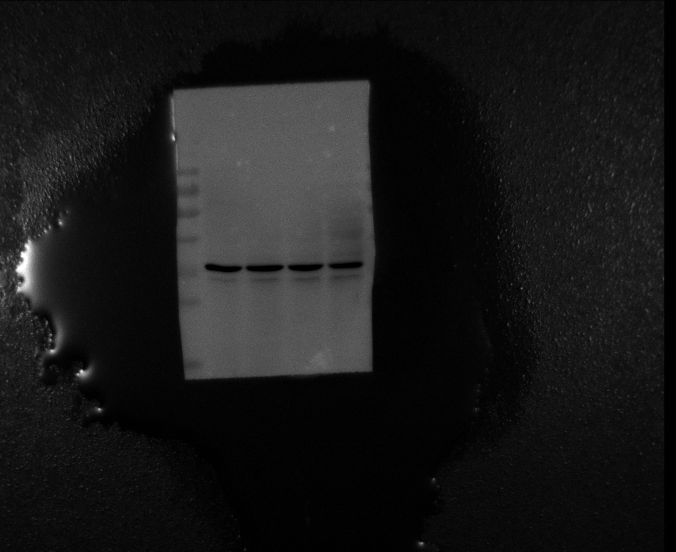

Supplement: Supplemental Information 1 [file peerj-11-15798-s001.zip › Uncropped GelsBlots/Figure 1/A/GAPDH original image.tif]

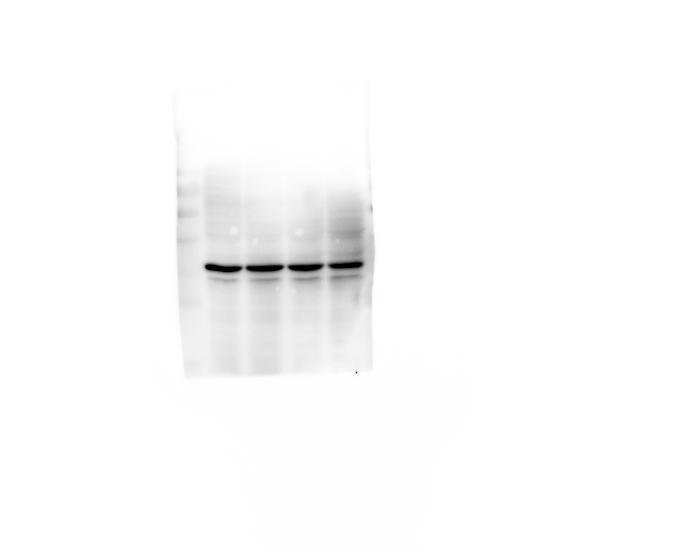

Supplement: Supplemental Information 1 [file peerj-11-15798-s001.zip › Uncropped GelsBlots/Figure 1/A/GAPDH.tif]

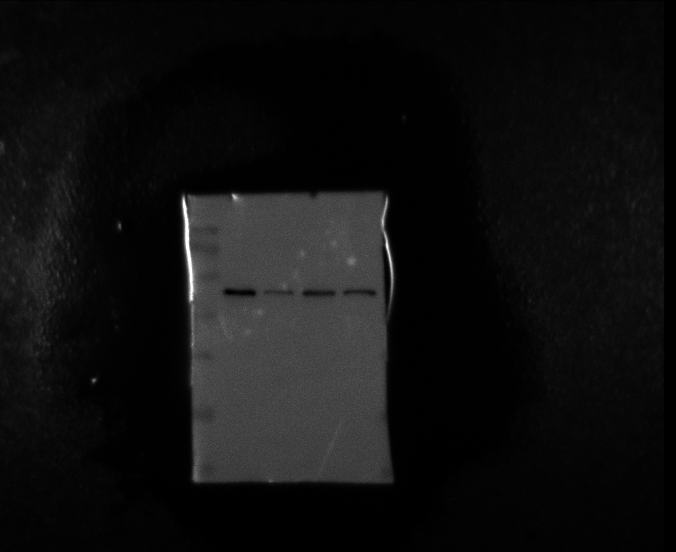

Supplement: Supplemental Information 1 [file peerj-11-15798-s001.zip › Uncropped GelsBlots/Figure 1/A/VDR original image.tif]

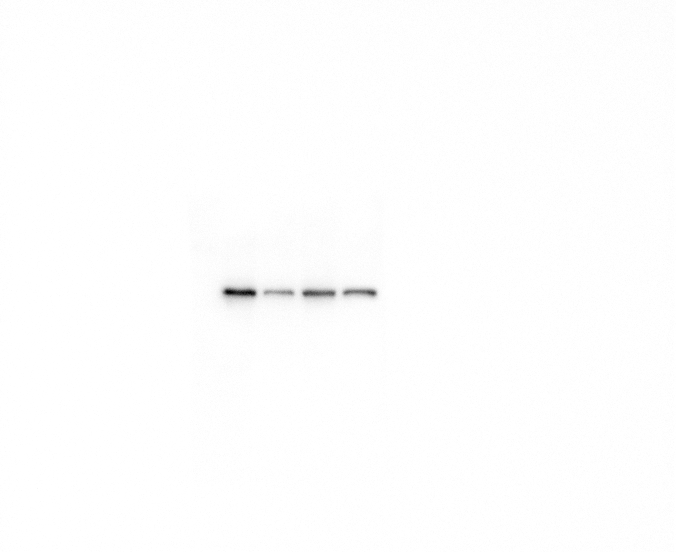

Supplement: Supplemental Information 1 [file peerj-11-15798-s001.zip › Uncropped GelsBlots/Figure 1/A/VDR.tif]

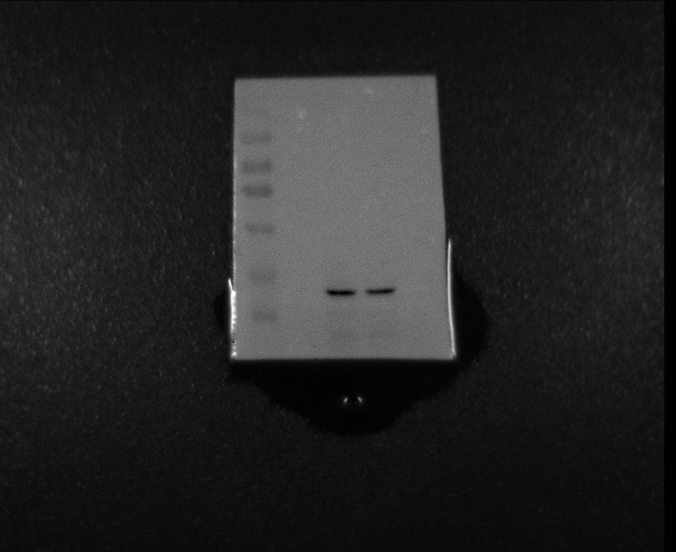

Supplement: Supplemental Information 1 [file peerj-11-15798-s001.zip › Uncropped GelsBlots/Figure 1/B/CD63 26KD original image.tif]

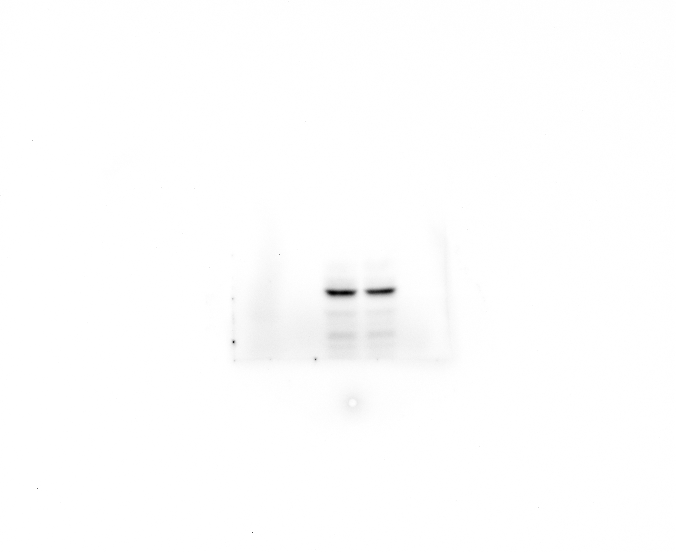

Supplement: Supplemental Information 1 [file peerj-11-15798-s001.zip › Uncropped GelsBlots/Figure 1/B/CD63.tif]

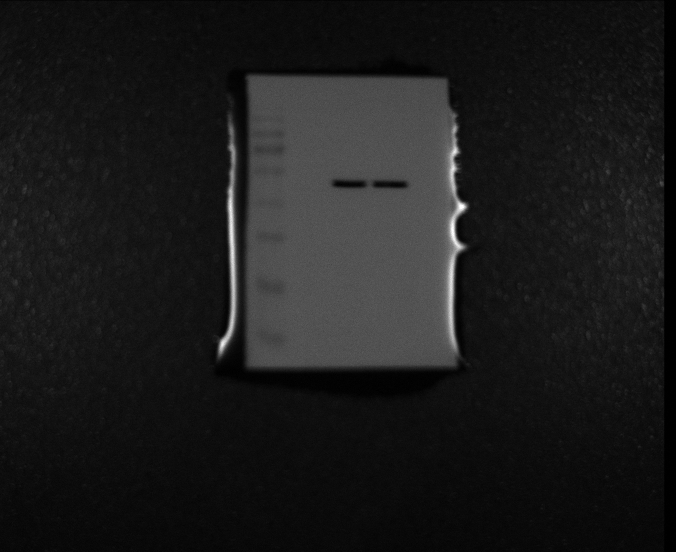

Supplement: Supplemental Information 1 [file peerj-11-15798-s001.zip › Uncropped GelsBlots/Figure 1/B/TSG101 44KD original image.tif]

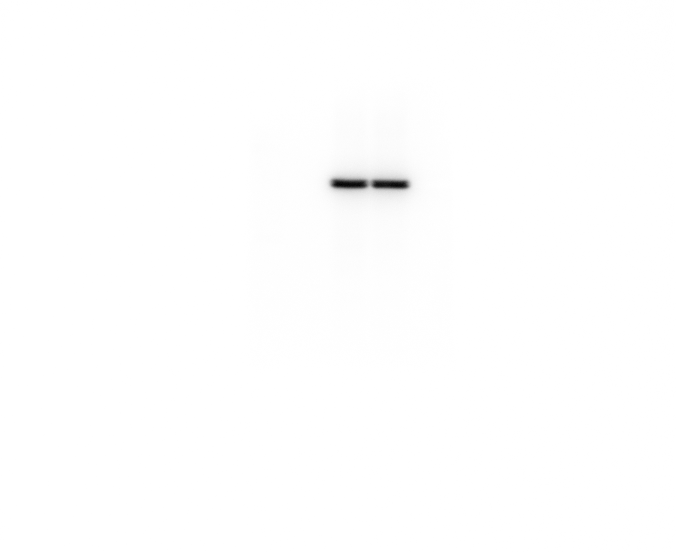

Supplement: Supplemental Information 1 [file peerj-11-15798-s001.zip › Uncropped GelsBlots/Figure 1/B/TSG101.tif]

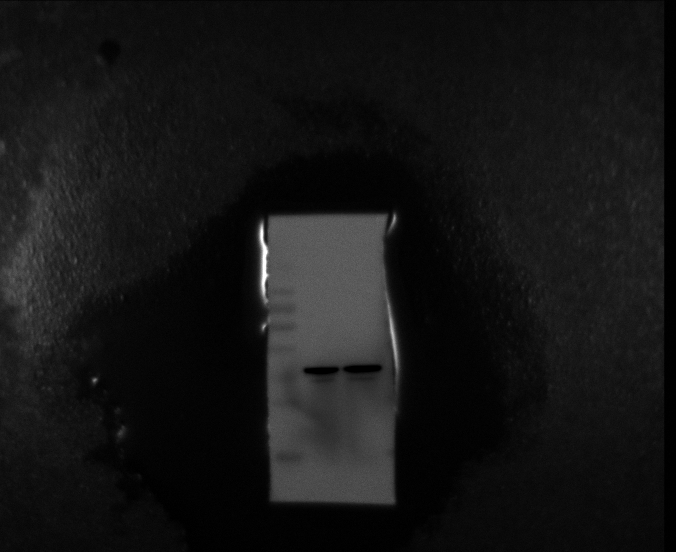

Supplement: Supplemental Information 1 [file peerj-11-15798-s001.zip › Uncropped GelsBlots/Figure 1/E/GAPDH original image.tif]

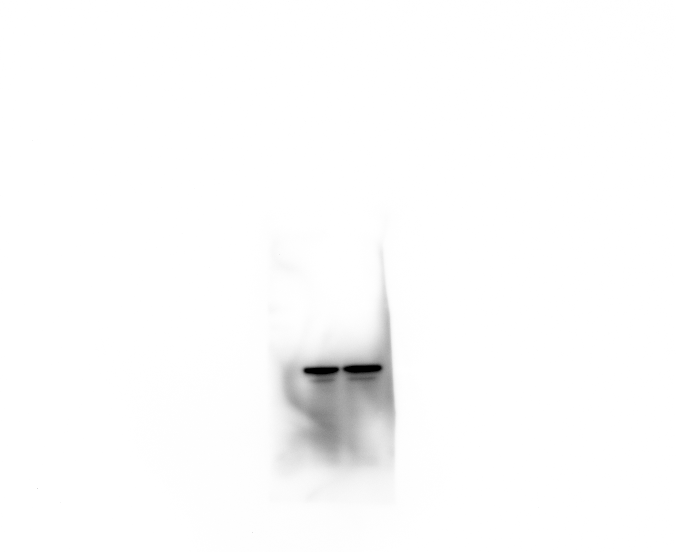

Supplement: Supplemental Information 1 [file peerj-11-15798-s001.zip › Uncropped GelsBlots/Figure 1/E/GAPDH.tif]

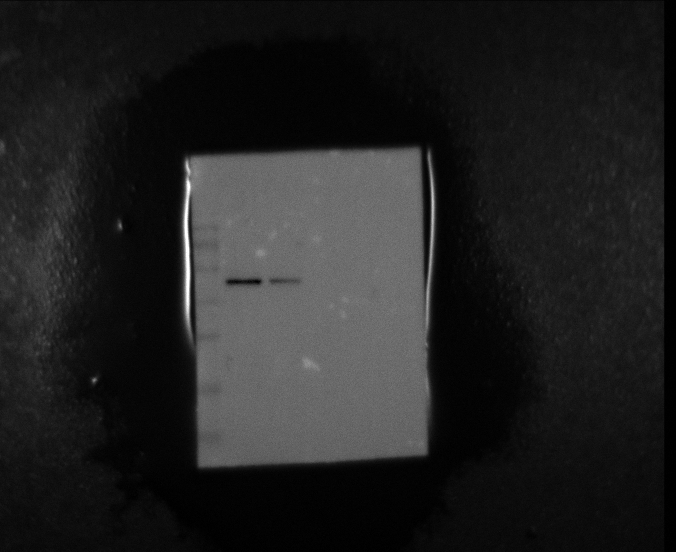

Supplement: Supplemental Information 1 [file peerj-11-15798-s001.zip › Uncropped GelsBlots/Figure 1/E/VDR original image.tif]

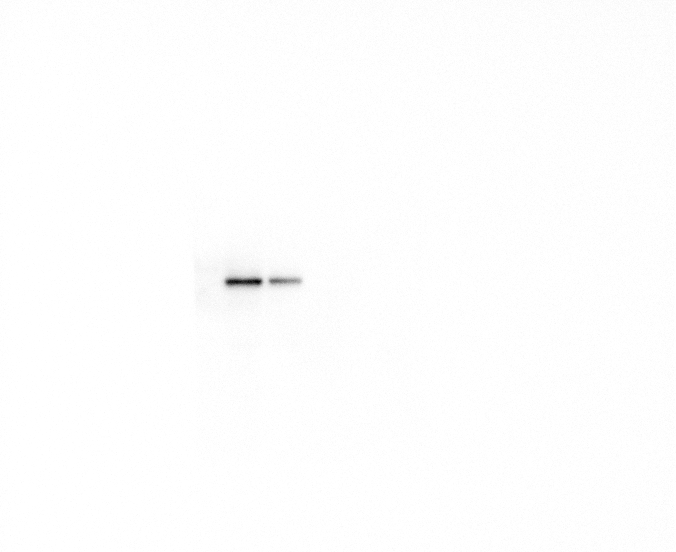

Supplement: Supplemental Information 1 [file peerj-11-15798-s001.zip › Uncropped GelsBlots/Figure 1/E/VDR.tif]
